# Daily functioning and (health-related) quality of life of young adult survivors of childhood bacterial meningitis

**DOI:** 10.1007/s00431-024-05819-6

**Published:** 2024-10-18

**Authors:** Omaima El Tahir, Rogier C. J. de Jonge, Jeroen Pronk, Sui Lin Goei, Caroline B. Terwee, A. Marceline Tutu van Furth

**Affiliations:** 1 Department of Pediatric Infectious Diseases and Immunology, AI&II, Amsterdam UMC, Vrije Universiteit Amsterdam, Amsterdam, The Netherlands; 2 Pediatric Intensive Care Unit, Departments of Pediatrics and Pediatric Surgery, Erasmus MC – Sophia Children’s Hospital, Rotterdam, The Netherlands; 3 Expertise Group Child Health, The Netherlands Organization for Applied Scientific Research (TNO), Leiden, The Netherlands; 4 LEARN! Learning Sciences, Faculty of Behavioral and Movement Sciences, Vrije Universiteit Amsterdam, Amsterdam, The Netherlands; 5 Department of Epidemiology and Data Science, Amsterdam UMC, Vrije Universiteit, Amsterdam Public Health Research Institute, Amsterdam, The Netherlands

**Keywords:** Quality of life, Bacterial meningitis, Long-term outcome

## Abstract

To investigate daily functioning, health-related quality of life (HRQoL), and overall quality of life (QoL) in young adult survivors of childhood bacterial meningitis (BM) thereby shedding light on very long-term outcomes and contributing to global meningitis burden estimates. In this cross-sectional study, the Weiss Functional Impairment Rating Scale-Self Report (WFIRS-S), the Patient-Reported Outcomes Measurement Information System Scale vGlobal Health v1.2 (PROMIS-GH), PROMIS-29 profile v2.01 and PROMIS Satisfaction with Social Roles and Activities Short Form 4a v2.0 instruments were administered to 483 survivors of childhood BM who participated in the 20|30 Dutch Postmeningitis study. Mean WFIRS-S scores were calculated as well as mean T-scores of the PROMIS instruments. PROMIS scores of survivors of childhood BM were compared with age-matched Dutch reference scores from the general population. Overall, survivors of childhood BM reported comparable or even better levels of daily functioning, HRQoL, and overall QoL (Global02) than the age-matched Dutch reference group. 10.2% of survivors of childhood BM reported poor mental health and 1.0% of survivors of childhood BM reported poor physical health. The average score of survivors of childhood BM for overall QoL (Global02) was 3.81 (on a scale from 1, indicating poor QoL, to 5, indicating excellent QoL).

*Conclusion*: Young adult survivors of childhood BM reported similar and even better levels of daily functioning, HRQoL, and overall QoL than age-matched Dutch reference scores. However, clinicians and healthcare workers should be mindful of poor mental and physical health of some survivors of childhood BM because these survivors also face problems with functioning in daily life.

**Table:** 

**What is Known:** • *Acute bacterial meningitis is a life-threatening infection which may lead to devastating lifelong disabilities.* • *Health-related quality of life is decreased in school-age survivors of bacterial meningitis.*
**What is New:** • *The present study shows that HRQoL in survivors of childhood BM improves over time* • *However, especially poor mental health in adolescent and young adult survivors is associated with impaired functioning in daily life.*

**Supplementary Information:**

The online version contains supplementary material available at 10.1007/s00431-024-05819-6.

## Introduction

Acute bacterial meningitis (BM) is a life-threatening infection of the central nervous system (CNS) with a peak incidence in neonates and the elderly. The disease has become less common in developed countries because of the widespread use of vaccines against *Haemophilus influenzae*, *Neisseria meningitides*, and *Streptococcus pneumoniae*, but still affects approximately five million people worldwide [1–3].The global burden of meningitis in all age groups remains high and progress lags substantially behind that of other vaccine preventable diseases [1]. From a call to action in 2017, the World Health Organization (WHO) developed a Defeating Meningitis by 2030 Global Roadmap in collaboration with important stakeholders such as the governments, health workers, and private sector representatives [3]. The roadmap contains a comprehensive vision for 2030 “Towards a world free of meningitis,” with three visionary goals: elimination of BM epidemics, reduction of cases of vaccine-preventable BM by 50% and deaths by 70%, reduction of disability, and improvement of quality of life (QoL) after meningitis due to any cause [3].

In the Netherlands, the incidence of BM decreased between 1989 and 2019 from 6.37 to 1.58 episodes per 100,000 population per year [4]. Currently, BM in the Netherlands is most commonly caused by *S. pneumoniae* with a mean annual incidence of 0.81 episodes per 100,000 population per year [4]. The incidence rates are highest in infants with 42.48 per 100,000 infants due to *Streptococcus agalactiae* and 19.49 per 100,000 infants for *Escherichia coli *[4]. BM can rapidly become fatal and lead to devastating lifelong disabilities in survivors, e.g., seizures, neuromotor disability, hearing and vision loss. As incidence rates are highest in infants, sequelae rates are also highest in infants with peak prevalent disability in this group [1]. Therefore, sequelae after childhood BM can persist and accumulate in adolescent and adult survivors of childhood BM [1]. Several studies have described the long-term outcomes of survivors of childhood BM [5–12]. A previous study by our research group with focus on survivors with mild sequelae showed decreased HRQoL in survivors of childhood BM, especially in survivors with intellectual and/or behavioral limitations, e.g., delay of language development, lower grade-point averages, problems with short-term memory or concentration, hyperactivity and impulsiveness [9, 13–15]. Furthermore, survivors may suffer from psychological distress and neurological sequelae, such as sensorineural hearing loss and seizure disorders [16]. Approximately half of the survivors of childhood BM suffer from long-term sequelae (follow-up time of at least five years) [16]. Despite all this research, studies investigating outcomes in adult survivors of childhood BM are scarce (follow-up of at least fifteen to twenty years) [16].

**Fig. 1:**
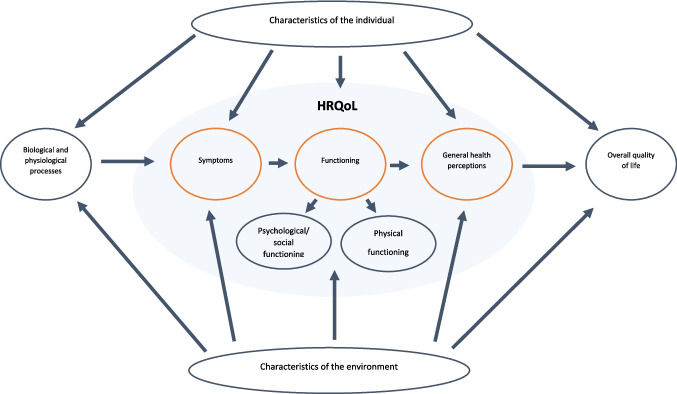
HRQoL model was adapted from Verbrugge and Jette [18], and Wilson and Cleary [19]

Therefore, the purpose of this study was to determine daily functioning, HRQoL, and overall QoL in young adult survivors of childhood BM and to compare HRQoL and overall QoL outcomes of survivors of childhood BM with representative data from the general Dutch population [17]. A conceptual model was used as a framework to study daily functioning, HRQoL, and overall QoL in line with the model described by Verbrugge and Jette and Wilson and Clearly (see Fig. 1) [18, 19]. In this model, a disease is defined in terms of disruptions in biological and physiological processes (e.g., infection and inflammation of the CNS related to BM). Disruptions in biological and physiological processes can lead to symptoms perceived by the patient (e.g., headache, nausea, fever, vision, and/or sensorineural hearing loss). These symptoms could lead to functional impairments in daily activities and affect general health perceptions and overall QoL. The term HRQoL was used to summarize the following outcome levels: (1) symptoms; (2) physical, psychological, and social functioning; and (3) general health perceptions (See Fig. 1). Overall QoL was defined as an overall general well-being that comprises objective descriptors and subjective evaluations (of physical, material, social, and emotional well-being together with the extent of personal development and purposeful activity, all weighted by a personal set of values) [20].

Our aim was not only to add to the literature on very long-term outcomes after childhood BM but also to provide input for global meningitis burden estimates. The cohort of survivors used in this study is one of the largest and longest follow-up cohorts of childhood BM survivors worldwide[21]. We hypothesized restrictions in daily functioning, and decreased HRQoL and overall QoL in young adult survivors of the childhood BM group, in line with previous research[9]. Moreover, previous research has shown variations in HRQoL within the group of childhood BM survivors. Specifically, the subgroup of survivors with academic and/or behavioral limitations showed the greatest decrease in QoL [9]. Therefore, potential differences in HRQoL within the group of childhood BM survivors were explored in the present study.

### Method

#### Sample and procedure

Two complete cohorts of 947 survivors of childhood BM were contacted and received a letter to participate in the present study as part of the Dutch 20|30 Postmeningitis study. The Dutch 20|30 Postmeningitis study was initiated to investigate long-term outcomes after childhood BM. In brief, the Dutch 20|30 Postmeningitis study investigated executive and behavioral functioning, mood and sleeping disorders, subjective hearing, HRQoL, academic performance, and economic self-sufficiency [21].

The present study investigates health-related quality of life and functioning in daily life. A large amount of data predicting quality of life was investigated in a separate article. The cohort survivors used in the Dutch 20|30 Postmeningitis study were composed of two independent, comparable historical cohorts [9, 21, 22]. For the first cohort, files from the Netherlands Reference Laboratory for BM (NRLBM) were searched in 1999 for children born between January 1986 and December 1994 who survived non-Haemophilus influenzae type b (Hib) BM between January 1990 and December 1995. For the second cohort, the NRLBM files were searched again in 2005 for Dutch children born between January 1993 and December 1999 who suffered from non-Haemophilus influenzae type b (Hib) BM between January 1997 and December 2001. The diagnosis of BM was based on the isolation of bacteria from cerebrospinal fluid (CSF). The exclusion criteria included a complex onset of meningitis (defined as meningitis secondary to immunodeficiency states, CNS surgery, cranial trauma, and CSF shunt infections of relapsing meningitis).

Data on daily functioning, HRQoL, and overall QoL in the present study were digitally collected between April 2018 and October 2019. A representative age-matched sample from the general Dutch population was included to compare HRQoL and overall QoL scores. The representative age-matched sample originates from a data collection company (Desan Research Solutions) recruited three waves of at least 1000 people from the Dutch general population from an existing internet panel in 2016. The panel was provided by Global Market Insite (GMI). Informed consent to become a panelist was obtained by GMI. Panelists were recruited by an invitation from the panel host to participate. By voluntarily responding to the invitation for this survey, panelists provided informed consent to participate in the study.

More details about the panel are provided by Elsman et al. [17]. The study samples (*N* = 1000–1300) were selected to be representative of the Dutch general population with respect to age distribution (18–40; 40–65; > 65), gender, educational level (low, middle, high), region of residence (north, east, south, west) and ethnicity (native Dutch, first- and second-generation western immigrant, first- and second-generation non-western immigrant) [17, 23–26]. The total study sample consisted of 4370 participants with mean age of 51 (range 18–93). Each study sample completed different items (e.g., Global Health, Pain Interference, Fatigue and Ability to Participate in Social Roles and Activities). For the present study, age-matched samples from the study samples (*N* = 1000–1300) with an 18–32 age range were selected. Forty-eight percent of participants were male. No reference group was available for the WFIRS-S scores. The medical ethics committee (MEC) of the VU University Medical Center Amsterdam, Amsterdam University Medical Center (AUMC) approved the Dutch 20|30 Postmeningitis study and written active consent was obtained from all participants.

### Daily functioning assessment

#### Weiss functional impairment rating scale-self report (WFIRS-S)

The WFIRS-S was used to measure daily functioning [27]. This instrument is an internationally used measure of ADHD-related functional impairment [27]. Good psychometric properties have been found in different countries, populations, settings, ages, and informants [27, 28]. The 69-item WFIRS-S assesses participants’ perspective of their own functioning across seven domains: Family (8 items), Work (11 items), School (10 items), Life skills (12 items), Self-concept (5 items), Social activities (9 items) and Risk activities (14 items). Each item is rated on the extent to which emotional or behavioral problems have impacted functioning in the last month on a 0–3-point Likert scale ranging from ‘not at all or never’ to ‘very much or very often’ and includes a ‘not applicable’ (NA) option [27]. The mean score allows for comparison between the domains. Using mean scores rather than the sum of the items ensures that items marked as NA are not included in the calculation of the overall score [27, 28]. The WFIRS was reported to have a high internal consistency with Cronbach’s alpha > 0.7 for each domain [29].

### HRQoL and overall QoL assessment

#### Patient-reported outcomes measurement information system (PROMIS.®)

PROMIS instruments were used to measure HRQoL and overall QoL [30–34]. PROMIS was initiated in 2004 by a collaboration of eight research centers and the United States National Institutes of Health [35]. The following features distinguish PROMIS instruments from other patient-reported outcome measures (PROMs) and make them preferable. These features include the use of item response theory and T-scores. Item response theory (IRT) models the relationship between the underlying trait being measured and the responses to items. For example, the higher a person’s level of functioning, the higher the probability that (s)he will report to be able to run five miles. The item “are you able to run 5 miles” is considered a relatively difficult item, while an item “are you able to get out of bed’ is considered an easy item. With IRT, the difficulty of all items (as well as their discriminative ability) in an item bank are established and used for calculating scores and for the selection of items for short forms. Most PROMIS measures in general use a T-score metric, where 50 is the mean of a relevant reference population and the standard deviation of this reference population is 10. PROMIS provides a web service called Scoring Service to calculate T-scores from respondents’ data based on the item parameters of the original US IRT model [36]. PROMIS T-scores for people aged 18–32 years from the general Dutch population (used as an age-matched reference group in the present study) were calculated based on five representative samples from the Dutch general population who completed the full PROMIS item banks in 2014–2016 [17].

The following PROMIS instruments were used in the present study to assess HRQoL and overall QoL:

#### PROMIS Global health scale

The Dutch version of the PROMIS-GH v1.2 consists of ten items: general health (Global 01), QoL (Global 02), physical health (Global 03), mental health (Global 04), social discretionary (Global 05), physical function (Global 06), pain intensity (Global 07), fatigue (Global 08), social roles (Global 09), and emotional problems (Global 10). This scale has been validated in a Dutch general population [14]. All items were rated on a 5-point Likert scale. The pain intensity item was scored on a 0–10 numeric rating scale ranging from “no pain” to “worst imaginable pain.” This last pain item was excluded from the present study because the PROMIS-29 Profile included the same item. For each item, a higher score indicates better health, except for Global08 and Global 10. Therefore, these items were reverse coded to calculate the scores. Global Mental Health (GMH) and Global Physical Health (GPH) subscales can be calculated, each of which includes four items. The GMH subscale includes the Global 02, Global 04, Global 05, and Global 10. The GPH subscale includes the Global 03, Global 06, Global 07, and Global 08. T-scores with a mean of 50 ± 10 (*SD*) were calculated for both the GMH and GPH subscales using the Scoring Service. The T-scores of childhood BM survivors were compared to those of age-matched individuals from the general Dutch population. To study the differences in health among childhood BM survivors, threshold values from the Dutch General population for poor, fair, good, very good, or excellent health calculated in the same study were used. A T-score of < 38 indicated poor GMH, a T-score between 38 and 42 indicated fair GMH, a T-score between 43 and 48 indicated good GMH, a T-score between 49 and 55 indicated very good GMH and a T-score ≥ 56 indicated excellent GMH. A T-score of < 35 indicated poor GPH, a T-score between 35 and 43 indicated fair GMH, a T-score between 44 and 50 indicated good GPH, a T-score between 51 and 56 indicated very good GPH and a T-score ≥ 57 indicated excellent GPH. The Cronbach’s alpha reported for GMH is 0.83, and 0.78 for GPH [37]. In this study, the Global 02 of the PROMIS-GH was used to assess the overall QoL of childhood BM survivors.

#### PROMIS-29 Profile

The Dutch-Flemish version of the PROMIS-29 profile v2.01 consists of twenty-nine items and assesses pain intensity using a single 0–10 numeric rating item and seven other domains of HRQOL (Pain, Fatigue, Sleep Disturbance, Anxiety, Depression, Pain Interference, Physical Function, and Ability to Participate in Social Roles and Activities). The PROMIS-29 has been validated in a Dutch general population sample [38]. Each health domain was measured using four items. Each item of the health domain is scored on a 5-point Likert scale [39]. There were five different 5-point Likert response scales: (1) ranging from unable to do to doing anything without any difficulty, (2) ranging from cannot do to do not at all, (3) ranging from always to never, (4) ranging from very bad to very good, and (5) ranging from very much to not at all. The PROMIS-29 scales were also scored as T-scores, with a mean of 50 ± 10 (*SD*) [35]. Higher scores indicated more of the domains measured (e.g., more pain or more function). Cronbach’s alpha ranged from 0.79 for Sleep Disturbance to 0.96 for Pain Interference [40].

#### PROMIS Satisfaction with the ability to participate in social roles and acitvities v2.0

The Dutch-Flemish version of the PROMIS Satisfaction with Social Roles and Activities Short Form 4a v2.0 consists of four items and assesses satisfaction with performing one’s usual social roles and activities. A short form was included in the present study to capture information on social functioning as an additional aspect of HRQoL. The item bank was validated in the Dutch general population [32]. All four items were scored on a 5-point Likert response scale ranging from very much to not at all. The short-form items were also scored as T-scores, with a mean of 50 ± 10 (*SD*). Higher scores indicated greater satisfaction. Cronbach’s alpha was reported high for all items (> 0.70) [41].

#### Statistical analyses

Descriptive statistics were used to describe the demographics and clinical characteristics of the participants and responses to the WFIRS-S and PROMIS items. Descriptive statistics were also used to calculate the mean T-scores for the poor, fair, good, very good, and excellent health subgroups. Categorical variables are presented as percentages (%); continuous variables are presented as mean (*SD*) or, when appropriate, as median (*IQR).* Independent *t*-tests were used to assess differences in average HRQoL and overall QoL scores (PROMIS-GH, PROMIS-29, PROMIS Satisfaction with Social Roles and Activities and Global 02 of PROMIS-GH) between survivors of childhood BM and the age-matched Dutch reference group. To correct for multiple comparisons, the Bonferroni procedure was applied to the independent *t*-tests. A significant *p*-value cut-off of 0.05/10 = 0.005 was applied using Bonferroni correction for the PROMIS-GH items. Significance cut-off of a *p*-value of 0.05/7 = 0.007 was applied using the Bonferroni correction for the PROMIS-29 Profile. Multiple regression analysis was performed to study associations between daily life functioning (WFIRS-S) and quality of life (PROMIS Global Health) adjusted for the following potential confounders: birth weight, administration of dexamethasone during infection, education level of parents, current age, age of onset, gender, causing pathogen. Unlike described in the study protocol gestational age and type of antibiotics were not included due to missing values. Education level of parents was used as indicator of socio-economic status. Focal neurological signs during admission or hospital stay were used to indicate severity of sequelae.

## Results

### Demographic characteristics

**Fig. 2:**
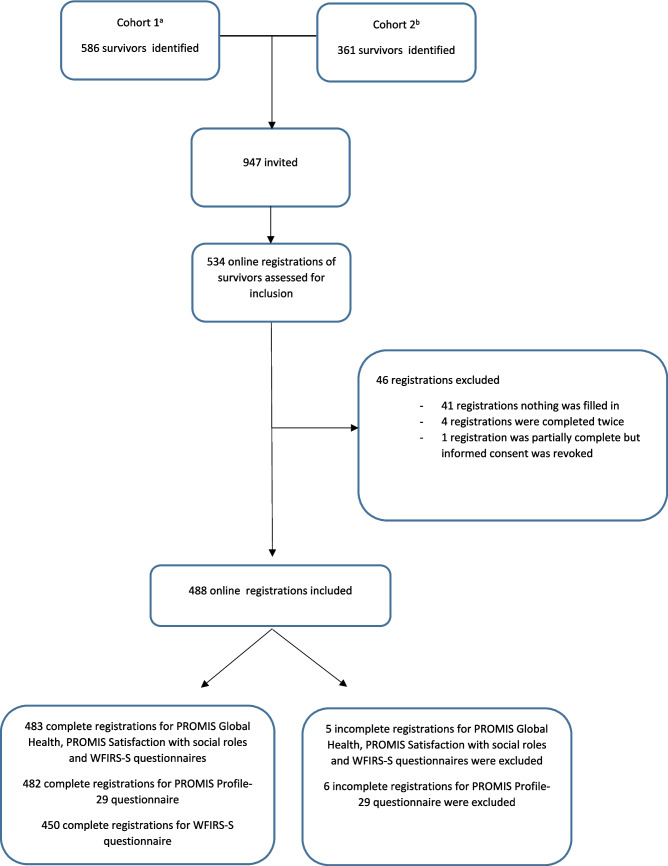
Flowchart demonstrating the selection of participants for the Dutch 20|30 Post meningitis study. ^a^ Koomen I, Grobbee DE, Jennekens-Schinkel A, Roord JJ, van Furth AM. (2003) Parental perception of educational, behavioural and general health problems in school-age survivors of bacterial meningitis. Acta Paediatr. 2003;92(2):177–85. ^b^ de Jonge RC, Sanders MS, Terwee CB, Heymans MW, Gemke RJ, Koomen I, et al. (2013) Unsuccessful validation of model for predicting academic or behavioural limitations after childhood bacterial meningitis. Acta Paediatr. 2013;102(12):e553-9

A total of 488 childhood BM survivors were included in the Dutch 20|30 post-meningitis study. After deduplication and exclusion of participants who did not complete any of the questionnaires, a subsample of 483 participants fully completed the PROMIS-GH and PROMIS Satisfaction with Social Roles and Activities. The PROMIS-29 Profile was fully completed by 482 childhood BM survivors, and the WFIRS-S was fully completed by 450 childhood BM survivors (see Fig. 2). The demographic and clinical characteristics of childhood BM survivors who participated in the present study are shown in Table 1. The differences in demographic and clinical characteristics between survivors of childhood BM who did not participate or were excluded in Dutch 20|30 post-meningitis study and the survivors of childhood BM included in the present study were not significant.

**Table 1:** Demographic and clinical characteristics of survivors of childhood BM

	Total sample *N* = 483
*Male, n (%)*	217 (45)
*Current age (years), median (IQR)*	26 (22–28)
*Time since meningitis (years), median (IQR)*	23 (19–25)
*Age at admission (months), median (IQR)*	25 (9–46)
*Low*^*a*^* educational level, n (%)*	297 (63)
*Low*^*a*^* educational level of parents, n (%)*	289 (60)
*Special needs in primary or secondary school, n (%)*	171 (36)
*Year repeated in primary or secondary school, n (%)*	111 (32)
*Employment status (unemployed), n (%)*	61 (13)
*Clinical characteristics*	
Pathogen, *n* (%)	
*Neisseria meningitidis*	368 (76.3)
*Streptococcus pneumoniae*	92 (19)
*E. coli*	6 (1.2)
*Streptococcus agalactiae*	16 (3.3)
*Listeria monocytogenes*	1 (0.2)
*Dexamethasone, n (%)*	106 (22)
*Symptoms* $>$ *72*^*b*^*, n (%)*	84 (17)
*Seizures, n (%)*	55 (16)
*Focal neurologic signs, n (%)*	31 (9)
*Hearing impairment*^*c*^*, n (%)*	41 (9)
*Disturbed consciousness*^*d*^*, (n (%)*	223 (66)
*CSF protein (g/L), (median IQR)*	1.35 (0.56–2.62)
*CSF glucose (mmol/L), (median IQR)*	1.75 (0.50–3.60)
*Ataxia, n (%)*	12 (3)

^a^Educational level was dichotomized as low (secondary and intermediate vocational education) and high (at least higher vocational education. ^b^Symptoms >  72 h before presentation. ^c^Hearing impairment was defined as unilateral or bilateral perceptive loss of  >  25 dB. ^d^Disturbed consciousness was observed during admission

One hundred-fifty participants from a previous study on HRQOL were included in the present study[9]. The mean HUI2 multi-attribute utility score was 0.89 (0.12), significantly lower score than the reference population used in the previous study 0.92 (0.08), *p* = 0.001. Overall QoL (Global02) score for this group was 3.81 (0.88) versus 3.2 (0.9) in the reference group (*p* =  < 0.001). The mean GMH score was 49.1 (8.58) versus 45.7 (8.1) in the reference group (*p* =  < 0.001) and mean GPH score was 50.9 (6.01) versus 48.1 (8.0) in the reference group (*p* =  < 0.001).

### Differences in HRQoL and overall QoL between survivors of childhood BM and the age-matched Dutch reference group scores

**Fig. 3:**
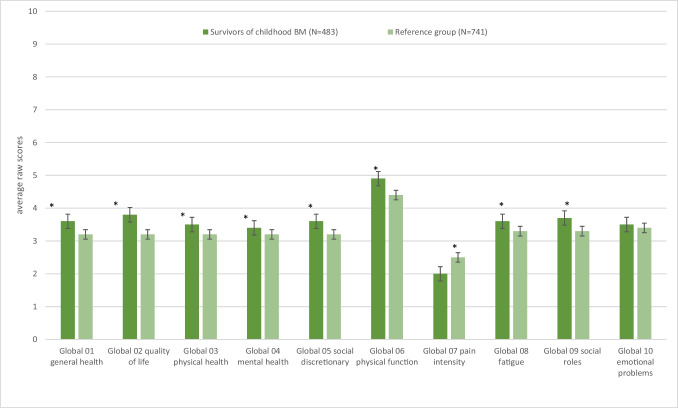
Average PROMIS Global Health scores of survivors of childhood BM and age-matched reference group. Differences between survivors of childhood BM and the reference group were significant (*p* = 0.001) for all Global Health items except for Global 10 (*p* = 1.000). All Global Health items were scored on a 5-point Likert scale, except Global07, which was scored on a 10-point Likert scale. Higher scores indicate more of the domain (e.g. more pain, better function)

**Fig. 4:**
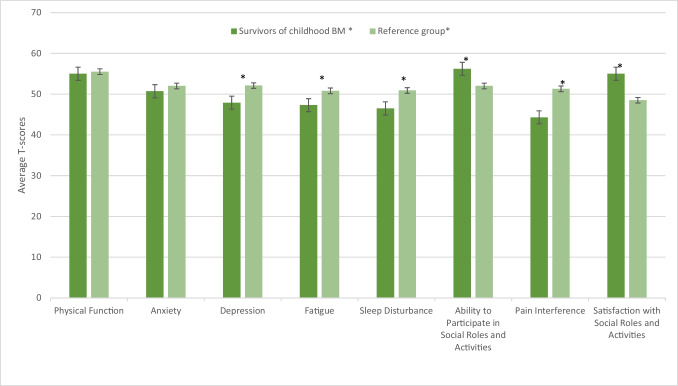
The average PROMIS-29 Profile and PROMIS Satisfaction with Social Roles and Activities T-scores of survivors of childhood BM and age-matched reference groups. *Sample sizes: PROMIS-29 Profile: survivors of childhood BM (*N* = 482), reference group: Physical Function (*N* = 242), Anxiety and Depression (*N* = 193), Fatigue and Sleep Disturbance (*N* = 162), Ability to Participate in Social Roles and Activities (*N* = 193), and Pain Interference (*N* = 159). PROMIS Satisfaction with Social Roles and Activities: survivors of childhood BM (*N* = 483) and the reference group (*N* = 178). Differences between survivors of childhood BM and the reference group were significant (*p* = 0.001) for all PROMIS-29 Profile domains except for Physical function (*p *= 0.33) and Anxiety (*p* = 0.12)

Figures 3 and 4 show the HRQoL and overall QoL (Global02) of childhood BM survivors compared to the age-matched reference group (see also the Supplementary information Table). For PROMIS-GH, the differences between survivors of childhood BM and the reference group were very small (0.1–0.6 T-score points), except for GMH (childhood BM survivors scored on average 3.4 points higher (better) than the reference group, 49.1 versus 45.7) and GPH (childhood BM survivors scored on average 2.7 T-score points higher (better) than the reference group, 50.8 versus 48.1). For PROMIS-29 Profile, the differences in scores between survivors of childhood BM and the reference group varied from 0.5 to 7.0 T-score points. Childhood BM survivors scored better on all PROMIS-29 domains except for the Physical Function domain. Thus, they had a lower average score on Depression, Anxiety, Fatigue, Sleep Disturbance, Pain Interference and a higher average score on Ability to Participate in Social Roles and Activities. For PROMIS Satisfaction with Social Roles and Activities, the difference in scores between the BM survivors and the reference group was 6.5 T-score points. Childhood BM scored higher (better) on Satisfaction with Social Roles than the reference group did.

**Fig. 5:**
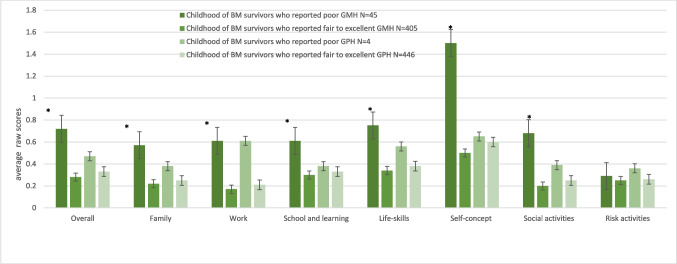
The average WFIRS-S scores of childhood BM survivors who reported poor GMH/GPH were compared to childhood BM survivors who reported fair to excellent GMH/GPH. *Significant differences in WFIRS-S domain scores between survivors of childhood BM who reported poor GMH and survivors who reported fair to excellent GMH. Significance cut-off of P-value 0.05/8  =  0.006 was applied using the Bonferroni correction

Overall mean daily functioning in the total group of survivors of childhood BM in the present study was 0.25 (SD 0.21). The group of survivors of childhood BM who reported poor GMH scored higher (worse) on all WFIRS-S domains compared with survivors of childhood BM who reported fair to excellent GMH (see Fig. 5). Thus, survivors of childhood BM who reported poor GMH experienced more problems in all the WFIRS-S domains. The differences in scores varied from 0.04 ( Risk activities) to 1.00 ( Self-concept). For GPH, the differences were smaller and varied from 0.04 (School and learning) to 0.40 (Work). The group of survivors of childhood BM who reported poor GPH scored higher (worse) than the group of survivors of childhood BM who reported fair to excellent GPH in all the WFIRS-S domains. Multivariable logistic regression models were performed to determine the association between GMH and WFIRS-S domains and GPH and WFIRS-S domains, adjusted for potentially confounding risk factors (See Table 2).

**Table 2:** Multivariable logistic regression assessing the association between GMH/GPH and overall WFIRS-S score

	Multivariable analysis OR (95% CI)	*P*-value
*GMH*^*a*^		
Overall score WFIRS-S	87.0 (23.1–328.0)	< 0.001
Gender	0.3 (0.3–1.5)	0.288
Causing pathogen	1.0 (0.7–1.4)	0.985
Birth weight	1.0 (0.9–1.5)	0.905
Administration of dexamethasone during infection	3.2 (0.9–10.6)	0.062
Education level of parents	0.6 (0.2–1.4)	0.231
Current age	1.1 (0.9–1.2)	0.280
Age of onset	1.0 (1.0–1.1)	0.536
Severity of sequelae^b^	3.2 (0.9–10.6)	0.062
*GPH*^*c*^		
Overall score WFIRS-S	11.8 (0.2–0.8)	0.090
Gender	4.4 (0.2–11.5)	0.600
Causing pathogen	1.3 (0.6–1.1)	0.003
Birth weight	1.0 (0.9–1.0)	0.487
Administration of dexamethasone during infection	3.0 ( 0.6–4.0)	0.995
Education level of parents	0.5 (0.2–0.7)	0.993
Current age	3.4 (0.6–19.6)	0.591
Age of onset	1.0 (0.9–1.1)	0.615
Severity of sequelae^b^	2.9 (2–12.5)	0.998

^a^Global Mental Health; ^b^Focal neurological signs during admission or hospital stay; ^c^*Global Physical Health*

## Discussion

The present study investigated the daily functioning, HRQoL, and overall QoL of young adult survivors of childhood BM. Survivors of childhood BM are at high risk of long-term neurologic and neuropsychologic deficits impairing daily life activities and quality of life [42, 43]. Insight in health and quality of life outcomes in the very long-term contributes to understanding in adolescent and adult survivors of childhood BM and manages expectations in parents of children with BM but also informs educators and healthcare professionals.

Strengths of this study are the high response rate of fifty-two percent approximately twenty-three years after illness and the age-matched Dutch reference group used to compare HRQoL and overall QoL scores. In general, the findings suggest comparable and even better levels of HRQoL and overall QoL in young adult survivors of childhood BM compared to the Dutch age-matched reference group. This finding was not in accordance with previous studies and our previously reported data that showed decreased HRQoL in postmeningitic children compared with a reference population of schoolchildren [5, 9, 11, 16]. However, different PROMs were used to collect data on HRQoL in those studies and as the children were younger, the measures were proxy-reported and might differ from the perspective of childhood BM survivors. No specific studies were performed to investigate the level of agreement between patient-reported and proxy-reported HRQol in childhood BM survivors, but other studies on self- versus proxy-reported paediatric HRQol showed mixed agreement between self-report and proxy-report [44–47]. Difference in follow-up duration is another important aspect that should be considered when evaluating the discrepancy in reported HRQoL. Sequelae and HRQol in childhood BM survivors might have partially recovered or improved over time [5, 6, 48].

Additionally, the disability paradox offers a potential explanation for the seemingly counterintuitive findings in the comparisons between the survivors of childhood BM and the age-matched reference group [49]. The disability paradox states that good or excellent QoL can be experienced by people with serious and persistent disabilities. The coherence model—part of the theoretical framework of the disability paradox—can be used to understand the discrepancy between perceptions of personal health and objective health status [49]. In the coherence model, good QoL from the viewpoint of people with disabilities reflects a reconstituted balance between the body, mind and spirit [49]. Similarly, poor QoL in these individuals reflects the absence of such a balance. Moreover, to the balance between body, mind, and spirit, emotions and the relationship of individuals to their social context and external environment may also explain the disability paradox [49]. When this perspective is applied to the findings of the present study, some young adult survivors of childhood BM seem to make sense of their disabilities (if present) and manage their lives in a better way. Thus, their glass is ‘half full’ which also applies to other pediatric study populations [50, 51].

Furthermore, the fact that survivors of childhood BM scored better than the age-matched reference group could be that the reference group compared themselves more strongly to others, while the survivors of childhood BM learned early in life that their condition makes it difficult for them to compare themselves to others. Therefore, survivors of childhood BM could be more easily satisfied with HRQoL and overall QoL. Disregarding the fact that the entire group of survivors of childhood BM scored better than the age-matched reference group, there were clear differences in reported HRQoL within the group of childhood BM survivors. The percentage of childhood BM survivors who reported poor mental health was higher than that of the survivors who reported poor physical health.

Differences in the results on most daily functioning (WFIRS-S) domains between the GMH and GPH subgroups of childhood BM survivors indicated more impaired daily functioning in the group of childhood BM survivors who reported poor GMH. Survivors of childhood BM who reported poor GMH especially reported problems on Self-concept, Life-skills and Social activities. For Risk activities, the differences between the GMH and GPH subgroups of childhood BM survivors were small. This might be explained by the average age of this study population and the fact that this domain was added to the instrument to capture serious but infrequent difficulties [19].

Our study has several limitations. First, problems regarding daily functioning, HRQoL, and overall QoL in survivors of childhood BM might have been underestimated due to selection bias. This might have occurred because we used an online testing procedure, which may have restricted the participation of patients with more severe sequelae. However, no significant differences in the number and type of sequelae at baseline were found between survivors of childhood BM included in the 20|30 Dutch Postmeningitis study and the remaining group of survivors who did not participate in the study (*p* = 1.000). No information was collected in this study on the actual minor or major sequelae in childhood BM survivors. On top of that, except for information on special needs regarding academic achievement during primary school no additional information on formally diagnosed psychiatric comorbidity and possibly corresponding physical and psychological therapies was not available or collected in survivors of childhood BM nor in the age-matched reference group from the Dutch population. Second, no information on changes and fluctuations in daily functioning, HRQoL, and overall QoL during adolescence and young adulthood was obtained because of the cross-sectional design of this study. Third, the WFIRS-S was initially developed to measure ADHD specific impairment but was used in the present study to assess daily functioning because of lacking common measures to assess daily functioning in survivors of childhood BM. Moreover, as the literature on very long-term outcomes after childhood BM is scarce, more information on daily functioning, HRQoL, and overall QoL of childhood BM survivors in the very long term is needed.

## Conclusion

The present study suggests good daily functioning, HRQoL, and overall QoL among young adult survivors of childhood BM. However, differences in daily functioning and HRQoL were found within the group of childhood BM survivors. Clinicians and healthcare workers should be mindful of poor mental health and impaired daily functioning later in life after childhood BM. In addition, they should acknowledge and recognize insufficient managing and coping capacities in survivors of childhood BM, because these survivors could potentially benefit from psychosocial support. Future studies should further assess persisting functional impairments later in life after childhood BM, as well as the needs of young adult survivors of childhood BM. In addition to the needs of survivors, more insight into prognostic factors that contribute to poor outcomes in young adults survivors of BM will serve as an input for more timely and supportive interventions.

## Supplementary Information

Below is the link to the electronic supplementary material.

Supplementary file1 (DOCX 17 KB)

## Data Availability

No datasets were generated or analysed during the current study.
