# Supplementary material for: Daily functioning and (health-related) quality of life of young adult survivors of childhood bacterial meningitis
Source: Eur J Pediatr. 2024 Oct 18;183(12):5441–53. doi: 10.1007/s00431-024-05819-6 (PMC11527968; doi:10.1007/s00431-024-05819-6)
Supplement: Supplementary file 1 — Supplementary file1 (DOCX 17 KB) [file 431_2024_5819_MOESM1_ESM.docx]

Table 1 *Average PROMIS scores of survivors of childhood BM compared to the age-matched reference group.*

|  | Individual Items | Survivors of childhood BM  *Mean* *(SD)* | Reference group^c^  *Mean* *(SD)* | Mean difference | 95% CI | *p*-value |
| --- | --- | --- | --- | --- | --- | --- |
| *PROMIS Global Health*  *(N=483) (N=741)^c^* | | | | | | |
|  | Global01 general health | 3.6 (0.8) | 3.2 (0.9) | 0.4 | 0.3 – 0.5 | 0.001^d^ |
|  | Global02 quality of life^a^ | 3.8 (0.8) | 3.2 (0.9) | 0.6 | 0.5 – 0.7 | 0.001^d^ |
|  | Global03 physical health^b^ | 3.5 (0.9) | 3.2 (0.9) | 0.3 | 0.2 – 4.5 | 0.001^d^ |
|  | Global04 mental health^a^ | 3.4 (1.0) | 3.2 (1.0) | 0.2 | 0.1 – 0.3 | 0.001^d^ |
|  | Global05 social discretionary | 3.6 (1.1) | 3.2 (1.0) | 0.4 | 0.3 – 0.5 | 0.001^d^ |
|  | Global06 physical function^b^ | 4.9 (0.5) | 4.4 (1.0) | 0.4 | 0.4 – 0.5 | 0.001^d^ |
|  | Global07 pain intensity^b^ | 2.0 (2.0) | 2.5 (2.5) | 0.4 | 0.2 – 0.7 | 0.001^d^ |
|  | Global08 fatigue^b^ | 3.6 (1.0) | 3.3 (1.0) | 0.3 | 0.4 – 1.7 | 0.001^d^ |
|  | Global09 ability to perform social roles | 3.7 (1.0) | 3.3 (0.9) | 0.4 | 0.3 – 0.5 | 0.001^d^ |
|  | Global10 emotional problems^a^ | 3.5 (1.0) | 3.4 (1.0) | 0.1 | 0.1 – 0.2 | 0.100^d^ |
|  | Global health subscales | | | | | |
| Global Mental Health | | 49.1 (8.6) | 45.7 (8.1) | 3.4 | 2.5 – 4.4 | 0.001^d^ |
| Global Physical Health | | 50.8 (6.0) | 48.1 (8.0) | 2.7 | 1.9 – 3.5 | 0.001^d^ |
| *PROMIS-29 Profile*  *(N=482) (N=242)^c^* | | | | | | |
|  | Physical Function | 55.0 (4.9) | 55.5 (9.4) | 0.51 | 0.5 – 1.6 | 0.334^e^ |
|  | *(N=482) (N=193)^c^* | | | | | |
|  | Anxiety | 50.7 (9.3) | 52.0 (9.9) | 1.3 | 0.3 – 2.9 | 0.118^e^ |
| *(N=482) (N=193)^c^* | | | | | | |
|  | Depression | 47.9 (8.4) | 52.1 (9.4) | 4.1 | 2.7 – 5.6 | 0.001^e^ |
| *(N=482) (N=162)^c^* | | | | | | |
|  | Fatigue | 47.3 (9.3) | 50.2 (6.0) | 2.9 | 1.2 – 4.5 | 0.001^e^ |
| *(N=482) (N=162)^c^* | | | | | | |
|  | Sleep Disturbance | 46.5 (7.6) | 50.9 (8.6) | 4.4 | 3.0 – 5.8 | 0.001^e^ |
| *(N=482) (N=193)^c^* | | | | | | |
|  | Ability to Participate in Social Roles and Activities | 56.2 (8.1) | 52.0 (8.8) | 4.2 | 2.7 – 5.6 | 0.001^e^ |
| *(N=482) (N=159)^c^* | | | | | | |
|  | Pain Interference^f^ | 44.3 (6.0) | 51.3 (8.8) | 7.0 | 5.8-8.2 | 0.001^e^ |
| *PROMIS Satisfaction with Social Roles and Activities*  *(N=483) (N=178)^c^* | | | | | | |
|  | Satisfaction with Social Roles and Activities | 55.0 (7.4) | 48.5 (7.9) | 6.5 | 7.8-5.2 | 0.001 |

*Notes.^a^items included in the Global Mental Health* component score; *^b^items included in the Global Physical Health scale; ^c^Sample sizes of the reference group differ per domain; ^d^Significance cut-off of 0.05/10=0.005 was applied using the Bonferroni correction ; ^e^Significance cut-off of 0.05/7=0.007 was applied using the Bonferroni correction, ^f^The pain interference item was rated on a ten-point Likert scale, all other items were rated on a five-point Likert scale.*
